# Pregabalin naproxencarbil in acute post-bunionectomy pain: a randomized clinical trial

**DOI:** 10.1097/PR9.0000000000001424

**Published:** 2026-01-20

**Authors:** Guang-Liang Jiang, J. Richard Evanson, Daneshvari Solanki, Dominick D'Aunno, Emanuel DeNoia, Juan Carlos Rondon, Alina Beaton, Louise Taber

**Affiliations:** a Xgene Pharmaceutical, Inc, Taipei City, Taiwan; b Legent Orthopedic Hospital, Carrollton, TX, USA; c First Surgical Hospital, Bellaire, TX, USA; d HD Research, Houston, TX, USA; e Endeavor Clinical Trials, San Antonio, TX, USA; f Clinical Pharmacology of Miami, Miami, FL, USA; g Pacific Research Network, San Diego, CA, USA; h Arizona Research Center, Phoenix, AZ, USA

**Keywords:** Acute pain, Postoperative pain, Naproxen, Pregabalin, Neuropathic pain, Inflammatory pain

## Abstract

**Introduction:**

Practice guidelines recommend multimodal approaches for better management of acute pain including the administration of two or more drugs that act by different analgesic mechanisms.

**Objective:**

The study aimed to assess pregabalin naproxencarbil (PNC), a new chemical entity targeting both nociceptive and neuropathic pain pathways, for the management of acute pain.

**Methods:**

This randomized, double-blind, placebo-controlled trial enrolled 450 participants scheduled for first metatarsal bunionectomy. Participants were randomized (1:1:1) to receive either 1250 mg or 750 mg of PNC or placebo. Study drug was administered 1 hour before surgery and thereafter every 12 hours for 72 hours. Participants reported numeric pain rating scale to calculate summed pain intensity (SPI). Rescue pain medication use and safety were monitored.

**Results:**

The least-squares mean (LSM) (SE) of SPI over 48 hours (SPI48) for the 1250 mg PNC group vs placebo as the primary efficacy end point was 132.6 (8.0) vs 286.0 (8.2) (*P* < 0.0001). For the 750 mg group vs placebo as the key secondary efficacy end point, the LSM (SE) of SPI48 was 157.9 (8.3) vs 289.5 (8.5) (*P* < 0.0001). Rescue medication was spared by 42.8%, 33.6%, and 2.7% participants in the 1250 mg, 750 mg, and placebo groups, respectively. Hazard ratios (95% confidence interval) for time to initiate rescue medication were 0.18 (0.14-0.25) for 1250 mg vs placebo and 0.24 (0.18-0.31) for 750 mg vs placebo. Tramadol consumption over 72 hours in the placebo was 4.1- or 3.0-fold higher than in the 1250 mg or 750 mg groups, and 3- or 2.5-fold higher with acetaminophen. Mild somnolence, dizziness, nausea, and constipation were more common in active groups.

**Conclusion:**

Pregabalin naproxencarbil dose-dependently reduced acute postoperative pain with acceptable safety profiles.

**Trial registration:**

ClinicalTrials.gov Identifier: NCT06017999.

## Abstract 2

Pregabalin naproxencarbil demonstrated analgesic effect and acceptable safety profiles in this postoperative acute pain trial.

Supplemental Digital Content is Available in the Text.

## 1. Introduction

Annually around 313 million surgeries are performed globally.^31^ In the United States, 86% of patients experience postoperative pain and 75% of them report moderate, severe, or extreme pain.^11^ Postoperative opioid use is 68%; and pain severity is associated with poor recovery and patient satisfaction.^16^ Inadequate control of acute pain can lead to chronic pain with significant morbidity, increased length of hospital stay, prolonged opioid use, and increased economic burdens.^5,10,12^ Hence, effective analgesia in the perioperative or traumatic period is a critical prophylactic step to prevent the progression to chronic pain.

Surgery and trauma inevitably involve the pathogenesis of local tissue inflammation, neural tissue damage, and inflammatory factor stimuli on neural terminals.^4,19,20^ Subsequent central pain sensitization and chronic neuropathic pain can develop if not managed well during the acute phase.^20^ Practice guidelines recommend multimodal approaches using analgesics with different mechanisms to match pain pathogenesis in managing acute pain.^2,6^

Pregabalin naproxencarbil (PNC) is a novel, nonopioid analgesic targeting both inflammatory and neuropathic pain signaling pathways. As a single molecule prodrug, it is not active in the gastrointestinal tract but metabolized into naproxen and pregabalin after absorption. Previous phase 1 trials in healthy volunteers demonstrated that pregabalin and naproxen derived from PNC had synchronized T_max_ values, and their C_trough_ levels increased by approximately 73% and 27%, respectively, compared with bioequivalent doses of pregabalin and naproxen taken as a fixed-dose combination ( Supplemental Fig. 1 and Supplemental Table 1, available at http://links.lww.com/PR9/A390). An earlier trial demonstrated the analgesic effects of PNC on chronic osteoarthritis pain with improved gastrointestinal safety.^21^ Here, we report the results of a phase 2b trial evaluating the efficacy and safety of PNC on acute pain in participants undergoing bunionectomy.

## 2. Methods

### 2.1. Participants

Participants aged 18 to 80 years, scheduled for unilateral first metatarsal bunionectomy at 8 US study sites, were enrolled if they met all inclusion criteria and none of the exclusion criteria. Participants had to be able to read and understand informed consent and questionnaires, and as well as communicate effectively with study staff. Participants were required to be nonpregnant, nonlactating, and use acceptable birth control. Participants had no contraindications to use nonsteroidal anti-inflammatory drugs or gabapentinoids. Participants with severe depression or anxiety were excluded. Subjects with concurrent painful conditions including chronic pain that may require analgesic treatment during the study period were excluded. No subjects were allowed to use other analgesics preoperatively, such as pain medications including: opioids were not allowed 2 days before surgery, corticosteroids were not allowed 14 days before surgery, selective serotonin reuptake inhibitors/serotonin-norepinephrine reuptake inhibitors were not allowed 7 days before surgery.

The trial was approved by Advarra Institutional Ethic Committee, Columbia, MD. The trial was designed and conducted in accordance with the International Council for Harmonisation Good Clinical Practice guidelines, the principles of the Declaration of Helsinki, and all relevant regulations. All participants gave written informed consent.

### 2.2. Trial design

This was a randomized, double-blind, placebo-controlled, parallel-group, dose-ranging, and multiple-center study (ClinicalTrials.gov Identifier: NCT06017999). An independent biostatistician generated and maintained randomization numbers using a computer program with a block size of 6 and study site was the stratification factor. A drug distributor at drug depot packaged and labeled study kits according to the randomization schedule and then delivered the kits to the pharmacy at each study site. Study drug and placebo were identical in appearance and smell and packaged identically except with different kit numbers. This biostatistician and the drug distributor concealed the randomization codes without any communication with research staff at sites or study operation team members until the database lock at the end of the study. Research staff at each site logged into an Interactive Web Response Systems with their specific username and password to acquire randomization number and corresponding kit number for a specific participant. The biostatistician was reachable to unblind a participant for safety reasons, if necessary, but was otherwise not involved in any other study activities. Participants and research staff responsible for assessments, monitoring, and data management were unaware of the participants' allocation throughout the trial.

Eligible participants were admitted to study centers the night before surgery and randomized in a 1:1:1 ratio to receive either 1250 mg PNC, 750 mg PNC, or matched placebo on the day of surgery. The study drug was administered starting 60 minutes before the bunionectomy, followed by doses every 12 hours for 72 hours. Participants were admitted to the study centers for 72 hours, where they were monitored and assessed by principal investigators and study staff. Participants were required to return to the clinic on day 15 as the exit visit.

Bunionectomy is a well-established bone pain model recognized by regulatory agencies for evaluating drug effects on acute pain. The procedure involves cutting of cutaneous tissues, a bone osteotomy, and screw fixation.^23^ It typically results in moderate-to-severe postoperative acute pain that is both inflammatory and neuropathic in nature.^25^ To ensure comparable surgical trauma across study sites, all participants underwent the Austin bunionectomy procedure with a Mayo block and sedation. The Mayo block was administered with up to 20 mL, 2.0% lidocaine without epinephrine. Propofol for sedation induction was at the discretion of the anesthesiologist with the intraoperative maintenance dose limited to 250 μg/kg/min. All participants were given fentanyl 50 μg and/or midazolam 1 mg intravenously at induction. The surgical procedure was limited to maximum duration of 90 minutes (the average surgical length in this trial was approximately 27 minutes).

All assessment schedules were anchored to the time of surgery completion, defined as the end of the last suture, also designated as hour “0” for subsequent assessments. Study staff recorded participant-reported pain levels using a standard 11-point numeric pain rating scale (NPRS) at the following timepoints postend of surgery: 0, 1, 2, 3, 4, 6, 8, 10, 12, 16, 20, 24, 30, 36, 42, 48, 54, 60, 66, and 72 hours within a ±10-minute window. The numeric pain rating scale was also collected immediately before administration of rescue or study medication. Patient global assessments of pain control (scale: poor, fair, good, very good, excellent) and sleep interference score (SIS) (0-10 numerical rating scale, 0 = no interference, 10 = worst interference) for the past 24 hours were collected at 24, 48, and 72 hours. Protocol-allowed pain rescue medications included intravenous acetaminophen 1 g, given every 6 hours *pro re nata* (PRN), up to 4 g within 24 hours. If a participant could not tolerate acetaminophen or experienced insufficient pain relief, 50 mg tramadol was administered every 4 hours PRN, up to 300 mg within 24 hours. If the rescue medications provided inadequate relief, the participants were discontinued from the study drug and transitioned to standard care while remaining in the trial for safety assessments. The time and the dose of rescue medication administered were recorded.

To minimize the contextual effect in the pain trial, clinical site staff were trained to interact with participants in a neutral manner. In addition, for consistency, participants were trained on how to accurately report their pain and complete other questionnaires before randomization.

### 2.3. Efficacy and safety measures

The primary efficacy end point was summed pain intensity (SPI) from end of surgery to 48 hours postsurgery (SPI48) for 1250 mg PNC vs placebo. The key secondary efficacy end point was SPI48 for 750 mg PNC vs placebo. Other secondary efficacy end points included median time to first use of rescue medication, total rescue medication consumption over 48 hours, patient global assessment at 48 hours, and SPI48 for the high dose PNC vs the low dose. Tertiary efficacy end points included the above efficacy measures at various timepoints up to 72 hours, SIS, and proportion of participants with rescue medication use at various timepoints.

Study staff recorded and monitored any adverse events (AEs), vital signs, and laboratory tests. Nausea was self-assessed using an 11-point numeric nausea rating scale (NNRS, 0 = no nausea, 10 = worst nausea possible) at 1, 4, 8, 12, 24, 48, 72 hours. Additional NNRS were collected if nausea or vomiting occurred or before any antiemetic administration. Respiration rate and oxygen saturation were also monitored. Sedation was assessed using the Ramsay sedation scale when NPRS was collected.

### 2.4. Statistical analyses

For sample size determination, based on an estimated standard effect size of 0.4,^24,26^ 130 evaluable subjects per group would yield 90% power at a 2-tailed α = 0.05 for the familywise type-1 error. The recruitment goal was set at 150 subjects per group. For multiplicity adjustment, a serial gatekeeping procedure was used to control the overall type-1 error rate (0.05) for the primary and the key secondary end point. The intent-to-treat (ITT) population including all randomized subjects was used for the primary analysis. Other populations included for sensitivity analyses were modified ITT (mITT) population, defined as all ITT subjects who received at least 1 dose of study medication and had at least 1 NPRS assessment postend of surgery; per-protocol population (PPS), defined as all mITT subjects who have no major protocol deviations; and observed data ITT population, defined as ITT population with minimal imputation for missing data. The safety analysis population included subjects who received at least 1 dose of study drug.

Summed pain intensity was calculated as the area under the curve of NPRS measurements from end of surgery through the hour of interest, using the standard trapezoidal rule. For a particular subject, if no measurements were collected within the window of nominal timepoint ± middle time between previous and next timepoint, then the nominal timepoint was considered missing. If more than 1 measurement were collected, then the measurement closest in absolute time to the nominal time was used. For area under the curve computations, all timepoints were used based on actual collection time, regardless of windowing. Missing data were imputed using the last-observation-carry-forward in general, except as specified for the intercurrent events described below. The imputation of missing NPRS values before the first nonmissing, such as at “0” hour that some participants were not clearly awaking from anesthesia, was conducted using the median of nonmissing observed pain scores from subjects randomized to the same treatment group at that timepoint. Pain scores after rescue medication use were imputed using the pain intensity score collected immediately before the use of rescue medication for the duration of 6 hours for using acetaminophen and 4 hours for using tramadol. If a second rescue medication was received before the end of the rescue window for the prior rescue administration, the rescue window and imputation were extended accounting for the maximum of the window of the 2 rescue administrations. Discontinuation from treatment due to AEs or lack of efficacy (LOE) would be imputed with worst-observation-carried forward. Receipt of any rescue medication outside of the protocol was processed as LOE. Missing data after study withdrawals for reasons other than LOE or AEs were treated as missing at random and imputed with means generated through multiple imputation model using 20 imputed data sets.

Least-squares mean (LSM) differences were calculated using an analysis of covariance model for hypothesis testing of SPI, NPRS, rescue medication consumption, and SIS. Treatment comparisons between 1250 mg PNC, 750 mg PNC, and placebo were pair-wisely compared as advised by US Food and Drug Administration. For each pairwise treatment comparison, separate models were used only with data from the treatments being compared. Hazard ratios were calculated from a Cox-Proportional Hazard model for testing of time to first rescue medication use. All *P* values and significance claims in figures and tables were based on LSM comparison from an analysis of covariance model, including treatment and site as fixed effects. Sensitivity analyses for SPI and NPRS measures were conducted with mITT, PPS, and observed data of ITT populations. Safety data were summarized descriptively.

## 3. Results

**Figure 1.**
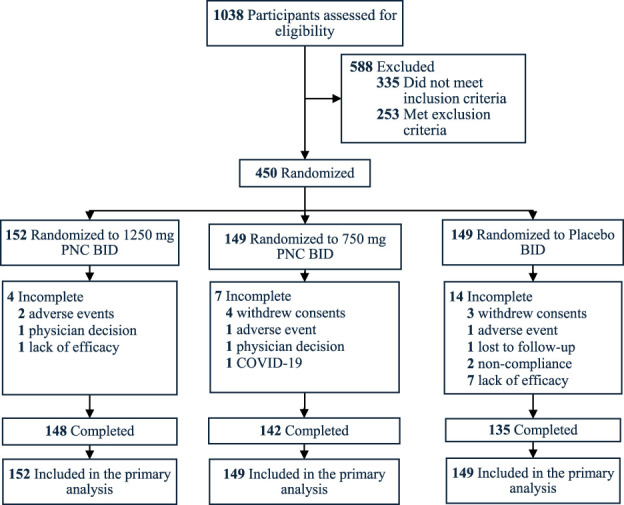
Recruitment, randomization, and follow-up. Primary analysis used intention-to-treat population including all randomized participants. PNC, pregabalin naproxencarbil.

A total of 450 qualified participants (median age, 48 years; 80% female) were recruited at 8 study sites across the United States of America from August 2023 to September 2024 (Fig. 1). All participants were included in the primary analysis, with 94.4% completing the 72-hour treatment (Fig. 1). Baseline demographic and clinical characteristics, such as age, sex, race, and BMI, were similar across treatment groups (Table 1).

**Table 1:** Demographics and baseline clinical characteristics of the primary analysis population.

Characteristics	1250 mg PNC (N = 152)	750 mg PNC (N = 149)	Placebo (N = 149)
Age, y, median (range)	45 (18-74)	50 (18-75)	48 (18-78)
Sex, n (%)			
Female	123 (80.9)	117 (78.5)	120 (80.5)
Male	29 (19.1)	32 (21.5)	29 (19.5)
Race, n (%)			
White	103 (67.8)	82 (55.0)	94 (63.1)
Black or African American	40 (26.3)	55 (36.9)	49 (32.9)
Asian	5 (3.3)	2 (1.3)	1 (0.7)
American Indian or Alaska Native	0	1 (0.7)	0
Native Hawaiian or other Pacific Islander	0	1 (0.7)	0
Unknown	0	0	1 (0.7)
Not reported	1 (0.7)	4 (2.7)	1 (0.7)
Other	1 (0.7)	3 (2.0)	3 (2.0)
Multiple	2 (1.3)	1 (0.7)	0
Ethnicity, n (%)			
Hispanic or Latino	57 (37.5)	50 (33.6)	57 (38.3)
Not Hispanic or Latino	95 (62.5)	97 (65.1)	91 (61.1)
Not reported	0	2 (1.3)	1 (0.7)
BMI, median (range)	28.3 (19.9-39.7)	27.9 (20.3-38.9)	27.7 (19.1-38.4)

Sex, race, and ethnicity were reported by participants.

BMI, body mass index; PNC, pregabalin naproxencarbil.

### 3.1. Primary and key secondary efficacy outcomes

The LSM (SE) for the primary efficacy end point in SPI48 for the 1250 mg PNC vs placebo was 132.6 (8.0) vs 286.0 (8.2), *P* < 0.0001, indicating a 53.6% reduction over the placebo group (Table 2). For the key secondary efficacy end point in SPI48, the LSM (SE) of the 750 mg group vs placebo was 157.9 (8.3) vs 289.5 (8.5), *P* < 0.0001, reflecting a 45.5% improvement over the placebo group (Table 2).

**Table 2:** Efficacy end points.

End point	1250 mg PNC LSM ± SE (N = 152)	750 mg PNC LSM ± SE (N = 149)	Placebo LSM ± SE (N = 149)	LSMD (95% CI)	*P*
Primary end point					
1250 mg vs placebo, SPI48	132.6 ± 8.0	NA	286.0 ± 8.2	−153.4 (−173.8 to −132.9)	<0.0001
Key secondary end point					
750 mg vs placebo, SPI48	NA	157.9 ± 8.3	289.5 ± 8.5	−131.6 (−153.1 to −110.2)	<0.0001
Other secondary end points					
1250 mg vs 750 mg, SPI48	135.4 ± 8.2	157.8 ± 8.2	NA	−22.4 (−43.5 to −1.3)	0.037
Tramadol use over 48 h (MEQ, mg)					
1250 mg vs placebo	4.4 ± 1.1	NA	17.4 ± 1.2	−13.0 (−16.0 to −10.1)	<0.0001
750 mg vs placebo	NA	6.0 ± 1.2	18.1 ± 1.2	−12.2 (−15.2 to −9.2)	<0.0001
Acetaminophen use over 48 h (mg)					
1250 mg vs placebo	1191.8 ± 148.5	NA	3776.0 ± 151.5	−2584.2 (−2965.2 to −2203.2)	<0.0001
750 mg vs placebo	NA	1540.5 ± 149.4	3836.3 ± 151.8	−2295.8 (−2682.1 to −1909.5)	<0.0001
Time to first use of rescue medication (h)					
Median (IQR)	31.5 (6.5 to NE)	12.2 (6.4 to NE)	4.0 (2.6 to 6.2)	NA	NA
Hazard ratio (95% CI)*	0.18 (0.14 to 0.25)	0.24 (0.18 to 0.31)		NA	<0.0001
PGA of pain control at 48 h, mean ± SD*	3.0 ± 1.1	2.9 ± 1.2	1.8 ± 1.2	NA	<0.0001
Tertiary efficacy end points					
SIS at various timepoints					
24 h	1.49 ± 0.3	NA	4.8 ± 0.3	−3.3 (−4.0 to −2.6)	<0.0001
	NA	2.0 ± 0.3	5.0 ± 0.3	−3.0 (−3.7 to −2.2)	<0.0001
48 h	1.2 ± 0.2	NA	3.4 ± 0.3	−2.2 (−2.8 to −1.6)	<0.0001
	NA	1.4 ± 0.2	3.5 ± 0.3	−2.0 (−2.7 to −1.4)	<0.0001
72 h	1.1 ± 0.2	NA	3.3 ± 0.2	−2.2 (−2.8 to −1.7)	<0.0001
	NA	1.6 ± 0.2	3.4 ± 0.2	−1.8 (−2.4 to −1.2)	<0.0001
PGA at various timepoints, mean ± SD*					
24 h	2.9 ± 1.0	2.8 ± 1.1	1.6 ± 1.2		<0.0001
72 h	3.2 ± 1.1	3.2 ± 1.0	2.0 ± 1.2		<0.0001
Risk difference over placebo in rescue medication users, % (95% CI)*					
0-24 h	−49.2 (−57.4 to −39.7)	−34.4 (−42.9 to −25.2)	NA	NA	<0.0001
24-48 h	−41.0 (−50.4 to −30.4)	−32.8 (−42.5 to −22.1)	NA	NA	<0.0001
48-72 h	−30.7 (−40.8 to −19.7)	−22.7 (−33.3 to −11.4)	NA	NA	<0.0001

LSM, least-squares mean; LSMD, least-squares mean difference; MEQ, morphine equivalent dose; NA, not applicable; NE, not estimable; PGA, Patient Global Assessment; PNC, pregabalin naproxencarbil; SIS, Sleep Interference Score; SPI, Summed Pain Intensity.

* *P* values were pair wisely compared with placebo, respectively.

### 3.2. Other secondary and tertiary efficacy outcomes

**Figure 2.**
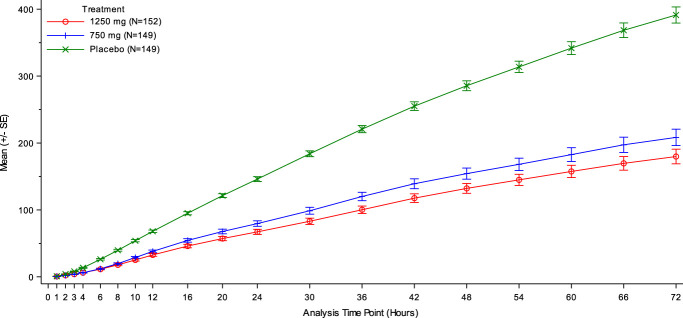
SPI over time. SPI is calculated from NPRS scores using the trapezoidal rule. The mean and standard error are calculated on the mean SPI from the same 20 imputed data sets. *P* < 0.0001 from 2 to 72 hours for each active group over placebo based on ANCOVA analysis. 1250 mg PNC was statistically significant from 750 mg PNC at each nominal timepoint from 16 to 54 hours, *P* < 0.05. ANCOVA, analysis of covariance; NPRS, numeric pain rating scale; PNC, pregabalin naproxencarbil; SPI, summed pain intensity.

The LSM differences of SPIs between 1250 mg PNC and 750 mg PNC were statistically significant at each nominal timepoint from 16 to 54 hours, with the 1250 mg group being lower (*P* < 0.05, Table 2 and Fig. 2). SPIs at each nominal timepoint from 2 to 72 hours were significantly lower in the PNC groups compared with the placebo group (*P* < 0.0001, Fig. 2).

**Figure 3.**
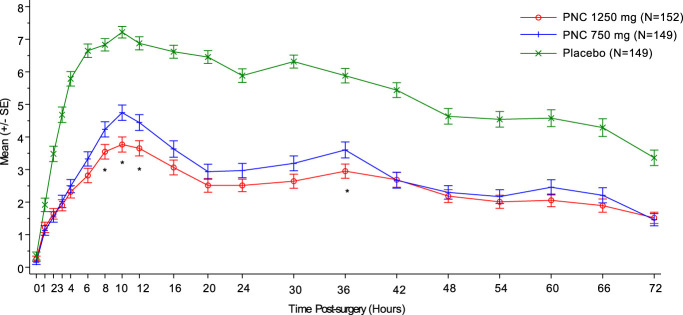
Pain intensity score over time. The mean and standard error were calculated on the mean NPRS score from 20 imputed data sets. *P* < 0.0001 to 0.05 from 1 to 72 hours for each active group over placebo based on ANCOVA analysis. *P < 0.05 for 1250 mg group vs 750 mg group based on least-squares mean estimates. ANCOVA, analysis of covariance; NPRS, numeric pain rating scale; PNC, pregabalin naproxencarbil.

Pain intensity measured by NPRS scores (Fig. 3) was statistically significantly lower in the PNC groups compared with the placebo from 1 to 72 hours (*P* = 0.0073 and 0.0014 for the high and low doses at 1 hour, respectively; and *P* < 0.0001 for the subsequent timepoints). At 8, 10, 12, and 36 hours, the 1250 mg PNC group demonstrated statistically significantly greater reductions in NPRS scores compared with the 750 mg PNC group (*P* < 0.05). Peak pain intensity in the placebo group (7.2 in the NPRS) was consistent with values reported in other bunionectomy studies, reaching moderate to severe levels.^14,22,28^ In comparison, the maximum mean pain score was mild (3.8 in the NPRS) in the 1250 mg PNC group. Sensitivity analyses on the above SPI and NPRS measures, using mITT, PPS, or observed data of the ITT population, demonstrated similar results.

**Figure 4.**
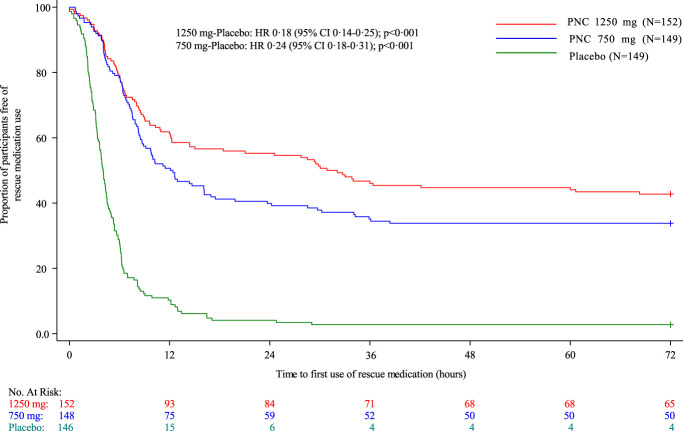
Kaplan–Meier plot on time to first use of rescue medication. Time to first use of rescue medication is computed as the first date-time of rescue medication minus the date-time of the end of surgery. Subjects who never used rescue medications or who had first use of rescue medication after 72 hours are censored at 72 hours. Four subjects that did not have surgery are not included in this figure. “+” indicates censored subjects.

The median time to first use of rescue medication was 31.5, 12.2, and 4.0 hours for the 1250 mg PNC, 750 mg PNC, and placebo groups, respectively (Table 2). The hazard ratios were 0.18 (95% confidence interval [CI], 0.14-0.25), *P* < 0.0001 for the 1250 mg group and 0.24 (95% CI, 0.18-0.31), *P* < 0.0001 for the 750 mg group, compared with placebo (Table 2 and Fig. 4). Over the 72-hour period, 42.8% (65/152) of participants in the 1250 mg PNC group, 33.6% (50/149) in the 750 mg PNC group, and 2.7% (4/149) in the placebo group did not use any rescue medication. The risk difference of subjects requiring rescue medication was statistically significant between active groups and placebo on each day of the 72 hours postsurgery (*P* < 0.0001, Table 2).

Tramadol and acetaminophen consumption over 48 hours was statistically significantly reduced in the 1250 mg and 750 mg groups relative to the placebo group (*P* < 0.0001, Table 2). Over 72 hours, tramadol consumption in the placebo was 4.1-fold higher than in the 1250 mg PNC group with LSM (SE) of 20.2 (1.5) vs 4.9 (1.5) mg (*P* < 0.0001), and 3.0-fold higher than in the 750 mg group with LSM (SE) of 21.2 (1.5) vs 7.2 (1.5) mg (*P* < 0.0001). Over 72 hours, LSM (SE) of acetaminophen use for the placebo vs the 1250 mg group was 4812.7 (219.1) vs 1586 (214.8) mg (3-fold, *P* < 0.0001), and vs the 750 mg group was 4892.7 (217.8) over 1975.9 (214.4) mg (2.5-fold, *P* < 0.0001).

Patient global assessment of pain control was statistically significantly higher in the PNC groups than in the placebo group at 24, 48, and 72 hours (*P* < 0.0001, Table 2). Sleep interference score of PNC groups were statistically significantly lower compared with the placebo group at 24, 48, and 72 hours postsurgery (*P* < 0.0001, Table 2). At 24 hours, sleep was moderately disrupted in the placebo group, whereas disruption was minimal in the PNC groups (Table 2).

### 3.3. Safety outcome

Investigators assessed all AEs without unblinding, and no serious AEs were reported. Mild adverse events were more common in the PNC groups than in the placebo group, with somnolence, dizziness, nausea, constipation, and vomiting being the most frequently reported (ie, ≥5%). Headache was reported slightly more in placebo group than in PNC groups. There was no difference in incidences of AEs with moderate or severe severity across groups. Overall, treatment-related or possibly related AEs were 28.9%, 20.1%, and 15.0% in the 1250 mg, 750 mg, and placebo groups, respectively (Table 3). Treatment discontinuation due to AEs were 3.3%, 2.0%, and 0.7% in 1250 mg PNC, 750 mg PNC, and placebo groups, respectively.

**Table 3:** Summary of adverse events.

Event*	1250 mg PNC (N = 152)	750 mg PNC (N = 149)	Placebo (N = 149)
Any adverse event, n (%)	88 (57.9)	69 (46.3)	62 (42.2)
Mild	79 (52.0)	60 (40.3)	51 (34.7)
Moderate	8 (5.3)	7 (4.7)	10 (6.8)
Severe	1 (0.7)	2 (1.3)	1 (0.7)
Serious adverse event, n (%)	0	0	0
Adverse events related or possibly related to study treatment, n (%)	44 (28.9)	30 (20.1)	22 (15.0)
Adverse event leading to treatment discontinuation, n (%)	5 (3.3)	3 (2.0)	1 (0.7)
Adverse events in ≥2% of participants in any group, n (%)			
Somnolence	23 (15.1)	16 (10.7)	0
Dizziness	21 (13.8)	17 (11.4)	6 (4.1)
Headache	7 (4.6)	8 (5.4)	12 (8.2)
Nausea	24 (15.8)	16 (10.7)	19 (12.9)
Constipation	18 (11.8)	13 (8.7)	7 (4.8)
Vomiting	9 (5.9)	5 (3.4)	6 (4.1)
Dry mouth	3 (2.0)	1 (0.7)	0
Dyspepsia	0	3 (2.0)	0
Alanine aminotransferase increased	3 (2.0)	3 (2.0)	1 (0.7)
Vision blurred	5 (3.3)	3 (2.0)	0
Diplopia	4 (2.6)	1 (0.7)	0
Hypertension	1 (0.7)	1 (0.7)	5 (3.4)
Hypotension	3 (2.0)	2 (1.3)	1 (0.7)
Fall	3 (2.0)	3 (2.0)	0
Rash	1 (0.7)	0	4 (2.7)
Anxiety	0	1 (0.7)	3 (2.0)

* Adverse events were classified according to Medical Dictionary for Regulatory Activities version 26.0 and graded according to Food and Drug Administration (FDA) Guidance on Toxicity Grading Scale for Healthy Adult and Adolescent Volunteers Enrolled in Preventive Vaccine Clinical Trials per FDA's instruction. Each participant was counted once, using the maximum severity of the adverse event.

The LSM (SE) of cumulative NNRS over 72 hours for 1250 mg PNC over placebo was 23.3 (5.3) vs 22.4 (5.6) (*P* > 0.05), and 14.2 (4.4) vs 21.8 (4.7) for 750 mg PNC over placebo (*P* > 0.05). Ramsay sedation scale scores were similarly rated across the groups as “cooperative, oriented, and tranquil” from 1 to 72 hours. In addition, no clinically significant issues were observed in laboratory tests, vital signs, standard 12-lead electrocardiograms, or physical examinations.

## 4. Discussion

This study demonstrated that PNC reduced acute bunionectomy pain in a dose-dependent manner. The high and low-dose PNC groups used approximately one-fourth and one-third of the tramadol compared with the placebo group, respectively. Similarly, acetaminophen consumption in the PNC groups was one-third to half of that in the placebo group. The median time to first use of rescue medication was delayed by 8-fold in the high-dose and 3-fold in the low-dose PNC groups, compared with placebo. Fewer participants required rescue medication in the active groups than in the placebo. Patient global assessment of pain control was improved with PNC treatment. PNC groups demonstrated reduced sleep interference relative to placebo.

From 2 to 72 hours, pain intensity reduced more than 50% in PNC groups (except hours 8, 10, and 12 for the low-dose group) compared to the placebo. There is no consensus on what constitutes a clinically meaningful between-group differences in acute pain. In chronic pain, an improvement of more than 30% is typically considered “much better” or a “clinically meaningful difference,” while an improvement of more than 50% represents a “substantial improvement.”^9,29^ Pregabalin alone has demonstrated opioid-sparing effects and pain relief at early hours but not later, in both bunionectomy and other postoperative pain trials.^15,24,30,32^ However, PNC increased the C_trough_ of pregabalin by approximately 73% compared with pregabalin dosed independently (supplement data, available at http://links.lww.com/PR9/A390). This formulation may help maintain therapeutic levels of pregabalin for a longer period, potentially prolonging its pain-relieving effects. The efficacy of PNC in this study may reflect its dual mechanism of action targeting both nociceptive and neuropathic pain arising from bunionectomy trauma. Naproxen blocks prostaglandin production, reducing inflammation and nociceptive nerve stimulation,^13,18^ while pregabalin decreases the hyperexcitability of peripheral and central neurons caused by nerve tissue injury or stimulation.^27^ In addition, naproxen and pregabalin from PNC had same T_max_ (ie, 4 hours in this trial), allowing their actions to synchronize, which may not be possible with a fixed-dose combination of pregabalin (T_max_ of 0.7-1.3 hours)^3^ and naproxen (T_max_ of 3 hours, based on our trials). Such a multiple-mode effect is not without precedent. Multimodal analgesia safely and effectively reduces postsurgical pain and opioid use.^1,12^ Even a single molecule, tapentadol, with dual mu-opioid receptor agonism and noradrenaline reuptake inhibition, also produced a robust analgesic effect with improved safety compared with oxycodone in bunionectomy trials.^7,8,23^

Mild somnolence and dizziness, known side effects of pregabalin, occurred at much lower rates than in other pregabalin trials.^17^ Somnolence side effects might have helped offset sleep disturbances postsurgery, as partially reflected by the improved SIS. Nausea, constipation, and vomiting were slightly higher in the PNC groups than in the placebo and are known side effects of naproxen.

A limitation of this study is its use of preemptive + treatment regimen rather than treatment regimen alone, because the onset of drug effect was uncertain before the trial. However, PNC treatment groups showed reduced pain scores compared with the placebo group 1 hour after surgery (the first timepoint for pain evaluation). Starting analgesics before elective traumatic procedures may reduce inflammation and prevent the development of peripheral and central sensitization, minimizing pain and the risk of developing chronic pain.^30^

## 5. Conclusion

Pregabalin naproxencarbil, a novel molecule that synchronizes inhibition of both inflammatory and neuropathic signals, reduced postoperative pain, opioid use, and sleep disturbance, with a clinically acceptable safety profile in this phase 2b trial. Its efficacy and safety in acute pain treatment await confirmation with further trials.

## Disclosures

G.-L. Jiang is an employee of Xgene Pharmaceutical Inc and possesses stocks of the company. L. Taber serves as a member of an Acute Pain Steering Committee for Vertex Pharmaceuticals since December 2023. The remaining authors have no conflicts of interest to declare.

## Supplemental digital content

Supplemental digital content associated with this article can be found online at http://links.lww.com/PR9/A390.
